# Physical Diagnosis1Read before the Pennsylvania State Veterinary Medical Association, September 3, 1895.

**Published:** 1895-10

**Authors:** Jacob Helmer

**Affiliations:** Scranton, Pa.


### PHYSICAL DIAGNOSIS.[1]

BY JACOB HELMER, D.V.S.
SCRANTON, PA.

PHYSICAL diagnosis is the process of distinguishing diseases by their physical (objective) signs, without recourse to subjective symptoms. It depends on scientific methods of physical examination, as inspection, auscultation, percussion, palpation, mensuration, and succussion, assisted by any mechanical aids or instruments that may, by extending the power of physical senses, be useful in detecting the signs of disease. Its conclusions are confirmed by clinical observation and the autopsy.

A paper limited to the time of a brief essay cannot apply the idea of this subject to the variety of diseases of the organs and tissues found in the animal body. We therefore select a group, the pulmonary diseases of the horse, and confine this article to a discrimination of them by the methods of physical diagnosis.

The value, comparatively, of the methods of physical examination is less in the veterinary than in the human practice of medicine. The anatomy of the horse's chest renders thorough physical examination extremely difficult. It is not easy to listen and hear sounds through such a thick wall. The voluntary movements of the skin over the panniculus carnosus muscle interfere. Some sound may not be heard, being under the scapular region. The usual restlessness of the animal and the awkward attitude of the examiner are among the obstacles to be overcome.

*Inspection.* This signifies the use of the sense of sight to detect physical signs. Inspection of the exterior of the chest reveals its form or shape and the number and character of its movements. We need not dwell upon the idea of form. It generally indicates in the horse degrees of vital capacity consistent with excellent health. Tubercular tendency or disease in the human family renders study of the form and size of the chest practical and necessary.

The movements of the chest are of great importance, since they constitute the first steps of the act of respiration, and all

[1] Read before the Pennsylvania State Veterinary Medical Association, September 3, 1895.

other steps depend on them. A respiratory act consists of an inspiration and expiration and a pause. This cycle is completed by a contraction and expansion of the chest-walls.

In health and at rest the movements of contraction and expansion are normal in number and character. When above normal number they are frequent, as during exercise. Frequency of movement not caused by exercise or thermic conditions is a sign of disease. Infrequent respiratory movements are abnormal and are usually due to morbid changes in the brain or the action of narcotic poisons.

The movements of the chest-walls may be partial or complete. A partial or limited movement of contraction signifies shallow respiration and the use of little air in the lungs. A complete or extended contraction indicates powerful and deep respirations and the change of much air in the lungs. These characters may be both frequent and infrequent.

The natural rhythm of the movements may be disturbed; hence these may be alternately regular and irregular. Again, the relation existing between the thoracic and abdominal movements in health may be broken. When by conditions of disease constriction of the thoracic muscles and diaphragm occurs, thus lessening their normal movement and amount of work, these movements in the assisting abdominal muscles becomes increased. This is well marked in pleurisy, where the abdominal muscles are brought to perform an unusual amount of work, while the thoracic muscles are more or less fixed on account of pain in the inflamed pleura. In peritonitis, where the pain is felt under the region of the abdominal muscles, these become fixed and the normal movements of the thoracic muscles are increased. Inspection in pleurisy with hydrothorax notes an increase in the size of the lower part of the chest. The attitude, breathing, and facial expression of the animal denote pain; the abdominal furrow or ridge the extent to which the abdominal muscles are brought into use. Inspection notes the double movements of the abdominal muscles in pulmonary emphysema, and assisted by the thermometer it notes the rise and fall of animal heat.

*Palpation.* This in medical language denotes the process of examining morbid conditions by the sense of feeling or by touch. By it we recognize tenderness, resistance, fluctuation, pain, the presence or absence of normal animal heat, also impulse from movements of an internal body or organ. Subcutaneous emphysema is detected by laying on the hand. It detects a crackling sensation under the skin caused by the movement of air in the cellular tissue. By palpation we note the character of tumors as fibrous, osseous, granular, soft, hard, or fluctuating. Palpation is useful as an aid to inspection and percussion. By it we note difference in resistance as well as of sound in percussion. Application of the hand to the chest informs us of the number and force of the beats of the heart, the shape of the chest-walls, their movements or number of respirations and the character of them. We note sensitiveness to pain on pressure, and sometimes the friction-murmur of pleurisy.

*Mensuration*, or a system of measurements of the chest as an aid to diagnosis, is not commonly practised by veterinarians. It might be useful in the study of pleurisy with hydrothorax in the horse, if the disease were unilateral as in man. But all we can learn by any system of measurements, however skilfully applied to the chest of the horse in pulmonary and cardiac trouble, may be known from the other methods. The first measurement is made with a graduated tape around the body from a point slightly posterior to the withers. At least three such measurements should be made at a few inches distant from each other. These measurements should be taken at the end of a complete inspiratory and expiratory movement; at least in the same stage of the respiratory movements. A second series of measurements are made beginning at a given point on the withers to the commencement of the cartilage of the false ribs, beginning with the eighth. Again, from the lower end of the third rib to the same points. Lastly, along the middle of the chest from the posterior border of the shoulder to the margin of the last rib. These measurements are made at the same points on both sides of the chest, and at the same point of contraction and expansion of the chest-walls.

*Auscultation and Percussion.* Of the methods of physical examination applied to detect disease of the chest the most important ones to us are ausculation and percussion. Before applying these methods let us for convenience and accuracy divide the chest into three regions, an anterior and two lateral. The lateral regions may be further subdivided in three nearly equal parts by drawing two horizontal lines across the chest. These subdivisions are a superior, a middle, and an inferior third. The superior third contains lung substances closely in contact with the internal wall. The middle third contains much lung substance; also the large bronchial tubes in its anterior part and the large bloodvessels at the base of the heart. In the lower third there is less lung substance, but in its anterior part the heart is principally situated opposite the third, fourth, fifth, and sixth ribs. This area, sometimes designated the triangular space, is covered by the forearm and its extensor muscles. The limb must be pulled forward in order to examine the region over the heart.

Auscultation is the art of listening over a part to detect abnormal sounds, and especially the condition that gives rise to them. It is the most important method of physical diagnosis. It was discovered by Reno Theophite H. Laennec, who lived from 1781 to 1826. It was introduced into veterinary practice by Delafond and Leblanc. The facts elicited by auscultation and percussion and their verification by post-mortem examination revolutionized the knowledge then in existence of the diseases of the chest and began an era of exact demonstration.

Auscultation is of two kinds, mediate and immediate. In the former an instrument, the stethoscope, is placed between the ear and the part to be examined. Immediate auscultation is when the ear is laid directly against the part. The veterinary practitioner should depend upon the ear only. An excuse for the use of a stethoscope is afforded when parts have been blistered, but a handkerchief may be laid between the ear and the part. To use the stethoscope successfully it is necessary to press the chest-piece quite hard against the parts, an action which the animal resists. Applied to the chest the object of auscultation is to discover sounds, normal or abnormal, in connection with the lung substance, pleura, or bronchi. It also notes absence of sound and sounds conducted from other parts.

The class of normal sounds heard over the lungs are bronchial breathing and the vesicular murmur. Bronchial breathing is normal when heard in the middle and upper third of the chest. The respiratory murmur is normal when heard over the middle portion and less distinctly over the superior and inferior thirds. Bronchial breathing sounds like a moderate current of air passing steadily through a tube or out of the nozzle of a bellows. It is closely imitated by making the sound of the letter K in the roof of the mouth. It is best heard over the anterior part of the chest at the bifurcation of the trachea. In health at the sides of the chest it is apt to be lost in the vesicular murmur.

The vesicular murmur is a sighing sound. It may be plainly heard over the chest of bovines or over the human chest. It is likened to the sound produced by the wind blowing steadily through green leaves. Bronchial breathing and the vesicular murmur are abnormal when heard in places other than in health. In pneumonia the vesicular murmur may be lost in the middle third and heard more plainly in the superior and inferior thirds of the chest. It then becomes an abnormal sound. Bronchial breathing is also an abnormal sound when it is heard plainly in the upper and lower third and lost in the middle third, due to blocking of that part of the lung with exudate. These two sounds may be increased or diminished in their normal situation. In pneumonia one part of the middle third may be blocked with exudate and the vesicular murmur lost in that part; but it will be found increased in other parts of the middle third not diseased.

We shall next consider the class of abnormal sounds known as râles. These are moist and dry. Moist râles are subdivided into mucous, submucous, and subcrepitant. Dry râles are divided into sonorous, crepitant, and sibilant. The sonorous râles are heard over the large bronchial tubes in the middle and upper portions of the chest in the first stage of bronchitis. The sibilant râle is heard in the upper and lower portions of the chest over the small bronchial tubes. It is a hissing sound. Bronchitis is a bilateral disease, hence these sounds may be heard over both sides of the chest, both on inspiration and expiration. The crepitant râle is heard in the first stage of pneumonia, principally over the middle portion of the lung. It is a snapping sound, and is closely imitated by stroking the hair of a cat in the opposite direction to which it lies. We may not see our patients early enough to hear this râle. It is caused by the air rushing into the air-cells and separating their walls, which had become more or less adherent by the exudate. Mucous râles are a bubbling sound heard in the upper portion of the chest on both sides. It sounds like minute bubbles due to blood, pus, or serum, or admixture of them. They are heard both on inspiration and expiration. If heard on the lower portion of the chest they are called submucous râles. These bubbling sounds change their place as the material moves along in the bronchial tubes. The subcrepitant râle is heard in croupous pneumonia. Here we have the exudate in the air-cells changing with liquid. The air passing in and out gives rise to a minute bubbling sound. It is heard in the third or resolving stage of pneumonia, both on inspiration and expiration.

Other Sounds: Gurgling sounds may be heard in the second stage of pneumonia, due to the movement of fluid in the large tubes, and may be heard on inspiration and expiration at the anterior part of the chest over the trachea and bronchi. Another sound is metallic tinkling. It is heard over the chest and at the nostrils in pleurisy during inspiration and expiration. It is not often heard at the chest. It is caused by drops of fluid falling into a collection on the bottom of the cavity. It may also be caused by the bursting of a bubble on the side of a cavity or by the imprisonment of a drop of water on the wall of a cavity. Metallic tinkling is pathognomonic in pleurisy.

Percussion is the process of striking a part with the tips of the bent fingers in order to elicit sounds, upon the character of which depends the condition of the tissues underneath. It was discovered by Leopald Avenbrugger, a Vienna physician, who lived from 1722 to 1809. He practised percussion only. He first began the study of sounds as indications of morbid conditions. The idea was introduced into France by Corvisart in 1821. Mediate percussion was suggested by Piorry in 1828. It is customary in percussion to lay the middle finger, or two fingers of the left hand, upon the part, and strike with the finger-tips of the right hand. Every physician has his own way of percussing. The striking should be a wrist movement, not a forearm movement. The blows should not be too heavy, and be made with discrimination. To percuss well is an art at which one can only become proficient by long practice. Sounds elicited by percussion vary in intensity, quality, duration, and pitch. Intensity means the loudness; *quality*, the kind of sound; *duration*, the length; and *pitch*, the degree of sound. Percussion-sounds are due to vibration of air in the air-cells and of the chest-walls. Percussion on a healthy subject shows that the results are not the same on both sides of the chest. Over the plane of the upper third, on the right side, the sound is the same to the eleventh rib back. Behind this there is a degree of tympanitic resonance. On the left side, over the upper third, the resonance gradually lessens from the twelfth rib back. Over the middle third, on both sides, the degree of resonance is about the same from the sixth to the twelfth rib. Behind this it is dull on right side and more resonant over the left side. On the lower third percussion is clear back to the tenth rib, where it becomes more dull. On the left side, dulness is found over the thirteenth rib back. So in the middle portion we find increasing pulmonary resonance, and in the superior and inferior portions we find diminished pulmonary resonance.

Resonance is divided into two kinds, pulmonary and tympanitic. Pulmonary resonance is that obtained by percussing over a healthy lung. Tympanitic resonance is heard when there is air inside of a cavity. It is a ringing metallic sound when caused in this manner. In percussion, sounds are increased, diminished, or absent. In the degree that it is diminished it is a dull sound. If absent it constitutes flatness. Flatness is due to effusion of fluid into a cavity. Pulmonary resonance is increased in the left lung if the right one is closed. As the sound depends on the amount of air in the tissues, when one part of the lung is blocked adjacent parts will become more resonant. Pulmonary resonance is diminished when there is less than a normal quantity of blood present, or where there is exudate, or where there is an increased amount of blood in a part, as in congestion of the lungs and in effusion into the interlobular connective tissue.

(To be continued.)

